# NHERF1/EBP50 and NF2 as diagnostic markers for choroid plexus tumors

**DOI:** 10.1186/s40478-016-0329-0

**Published:** 2016-05-27

**Authors:** Maria-Magdalena Georgescu, Bret C. Mobley, Brent A. Orr, Ping Shang, Norman L. Lehman, Xiaoping Zhu, Thomas J. O’Neill, Veena Rajaram, Kimmo J. Hatanpaa, Charles F. Timmons, Jack M. Raisanen

**Affiliations:** Department of Pathology, The University of Texas Southwestern Medical Center, Dallas, TX 75390 USA; Department of Pathology, Vanderbilt University Medical Center, Nashville, TN 37232 USA; Department of Pathology, St. Jude Children’s Research Hospital, Memphis, TN 38105 USA; Department of Pathology, The Ohio State University, Columbus, OH 43210 USA; Department of Neuro-Oncology, The University of Texas MD Anderson Cancer Center, Houston, TX 77030 USA; Department of Radiology, The University of Texas Southwestern Medical Center, Dallas, TX 75390 USA

**Keywords:** NHERF1/EBP50, NF2, Moesin, Choroid plexus tumors, Papillary tumor of the pineal region, CNS metastases

## Abstract

The adaptor protein NHERF1 (Na/H exchanger-3 regulatory factor-1) and its associated ezrin-radixin-moesin-merlin/neurofibromin-2 (ERM-NF2) family proteins are required for epithelial morphogenesis and have been implicated in cancer progression. NHERF1 is expressed in ependymal cells and constitutes a highly sensitive diagnostic marker for ependymoma, where it labels membrane polarity structures. Since NHERF1 and ERM-NF2 proteins show polarized expression in choroid plexus (CP) cells, we tested their diagnostic utility in CP neoplasms. NHERF1 immunohistochemistry in 43 adult and pediatric tumors with papillary morphology revealed strong apical plasma membrane staining in CP papilloma (WHO grade I) and cytoplasmic expression in CP carcinoma (WHO grade III). Ezrin and moesin showed similar but less distinctive staining. NHERF1 also labeled papillary tumors of the pineal region in a microlumen and focal apical membrane pattern, suggestive of a transitional morphology between CP papilloma and ependymoma. CP tumors of all grades could be differentiated from metastatic carcinomas with papillary architecture by NF2, which showed polarized membranous staining in CP tumors. NHERF1 and NF2 immunohistochemistry showed enhanced sensitivity and specificity for CP tumors compared to commonly used markers, including cytokeratins and Kir7.1, emerging as reliable diagnostic tools for the differential diagnosis of papillary tumors of the central nervous system.

**Electronic supplementary material:**

The online version of this article (doi:10.1186/s40478-016-0329-0) contains supplementary material, which is available to authorized users.

## Introduction

Choroid plexus (CP) cells are specialized polarized neuroepithelial cells that secrete the cerebrospinal fluid (CSF) and line the intraventricular papillae forming the CP [1]. These cuboidal cells have domed apical plasma membrane rich in microvilli, basement membrane at the basal pole, and are connected laterally by tight junctions. Similarly to epithelial cells of other origins, CP cells express cytokeratins. Due to active secretory function, over 65 % of the nearly 400 solute carrier transporters are expressed by the CP epithelial cells. NHERF1/EBP50 (Na^+^/H^+^ exchanger 3 regulating factor 1; ezrin-radixin-moesin (ERM) binding phosphoprotein 50) is an adaptor protein that interacts with solute carriers, such as Na^+^/H^+^ exchanger 3, and many other signaling molecules through its amino (N)-terminal PDZ (PSD95-Dlg1-ZO1) domains [2, 3]. It also interacts thourgh its carboxyl (C)-terminal ERM-binding region with the four members of the ERM-NF2 family of cytoskeletal proteins: ezrin, radixin, moesin and merlin [4]. The latter is the product of the neurofibromin 2 (*NF2*) gene, and will be called NF2 throughout. NHERF1 and ERM members form complexes at the plasma membrane required for epithelial morphogenesis and apical plasma membrane organization [5, 6]. Moreover, *NHERF1* knockout mice have structural defects in various specialized membranes bearing microvilli or cilia that lead to functional deficits [7–11].

Beyond their morphogenetic rolce, both NHERF1 and ERM-NF2 family members are implicated in tumorigenesis. Mutations of the tumor suppressor *NF2* cause a nervous system cancer predisposition syndrome, neurofibromatosis type 2 [2, 12, 13]. A tumor suppressor role has also been proposed for NHERF1, when localized at the membrane, through effects of the membrane-localized adaptor on its PTEN and β-catenin ligands [14–16]. Importantly, NHERF1 loss or displacement from the plasma membrane has been reported in aggressive tumors, including carcinomas and glioblastoma [14, 17, 18].

Due to their structural function and role in tumorigenesis, we analyzed the expression of NHERF1 and ERM-NF2 in normal CP and CP tumors. CP tumors arise most often in the pediatric population and, according to the 2007 World Health Organization (WHO) classification of central nervous system (CNS) tumors, they fall into three categories, in increasing order of aggressiveness: CP papilloma (WHO grade I), atypical CP papilloma (WHO grade II), where an increased mitotic index of 2 mitoses/10 high-power fields is a suggested diagnostic criterium, and CP carcinoma (WHO grade III) characterized by the presence of compact growth, necrosis, increased cellular density, nuclear pleomorphism, and more than 5 mitoses/10 high-power fields. Interestingly, we found that the distribution of NHERF1 changed with progression through the CP transformation spectrum: NHERF1 shifted from the apical plasma membrane in papilloma to the cytoplasm in carcinoma. This shift is not only compatible with a previously demonstrated tumor suppressor role for membrane-localized NHERF1, but can be used as diagnostic marker for distinguishing papillomas from carcinomas. Although rare in adults, the CP tumors do occur in this population and can pose a differential diagnostic dilemma with metastatic disease. We found that the distribution of NF2 can distinguish between CP tumors and papillary metastases, and we endorse using a combination of NHERF1 and NF2 immunohistochemistry (IHC) to address the differential diagnosis for papillary tumors of the CNS.

## Materials and methods

### Human specimens

Formalin-fixed paraffin-embedded brain tumor resection specimens were obtained from the Departments of Pathology of the University of Texas Southwestern Medical Center, Dallas, TX, Vanderbilt University, Nashville, TN, St. Jude Children’s Research Hospital, Memphis, TN, and the Ohio State University, Columbus, OH. Fresh normal CP from autopsy was obtained from the Department of Pathology, University of Texas Southwestern Medical Center, Dallas, TX. These studies were performed in compliance with the ethical guidelines of the Helsinki Declaration and approved by the ethical committees for research on human subjects of the institutions above mentioned.

### Histology, IHC and imaging

The specimens were processed for H& E staining and IHC as described [8], with antibodies for NHERF1 1:3200 (Thermo/Fisher, Waltham, MA), moesin 1:100 (3150, Cell Signaling Technology, Danvers, MA), Kir7.1 1:200, NF2 C-terminal 1:800 (C18) (see also [19]) (Santa Cruz Biotechnology, Santa Cruz, CA) and ezrin 1:400 (30252, BD Biosciences, San Jose, CA). Two certified neuropathologists reviewed independently the IHC results. Images were acquired and analyzed at various magnifications with an Aperio Scanscope CS2 whole slide image system (Leica Biosystems, San Diego, CA).

### Protein analysis

Freshly harvested CP was snap frozen, minced and Dounce-homogenized in ice-cold TNN lysis buffer [50 mM Tris HCl (pH 7.4)/150 mM NaCl/5 mM EDTA/0.5 % Nonidet P-40] containing proteinase inhibitor cocktail (Roche, Basel, Switzerland). After centrifugation, the protein concentration of the supernantant was measured and lysates with equal protein amounts were processed for Western Blot (WB) as described [20]. Antibodies used were: radixin (C15, Santa Cruz Biotechnology), actin (Chemicon/Millipore, Billerica, MA) and NHERF1, ezrin and moesin, as mentioned above for IHC. LN229 glioblastoma cell lysates expressing small hairpin (sh) RNA for ezrin, radixin and moesin were prepared as described [20].

## Results

### NHERF1 and NF2 have distinctive expression patterns in CP tumors

**Fig. 1:**
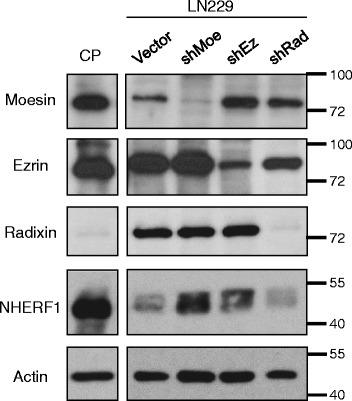
ERM protein detection in CP. WB analysis of human normal CP tissue lysate (30 μg proteins) with individual ERM and NHERF1 antibodies. Cell lysates from human LN229 glioblastoma cells infected with retroviruses carrying vector control and ERM specific shRNAs (Moe – moesin, Ez – ezrin, Rad – radixin) were run in parallel to test the specificity of the antibodies. Actin is used as loading control. Note specific detection of each ERM protein and lack of cross-reactivity between antibodies

**Fig. 2:**
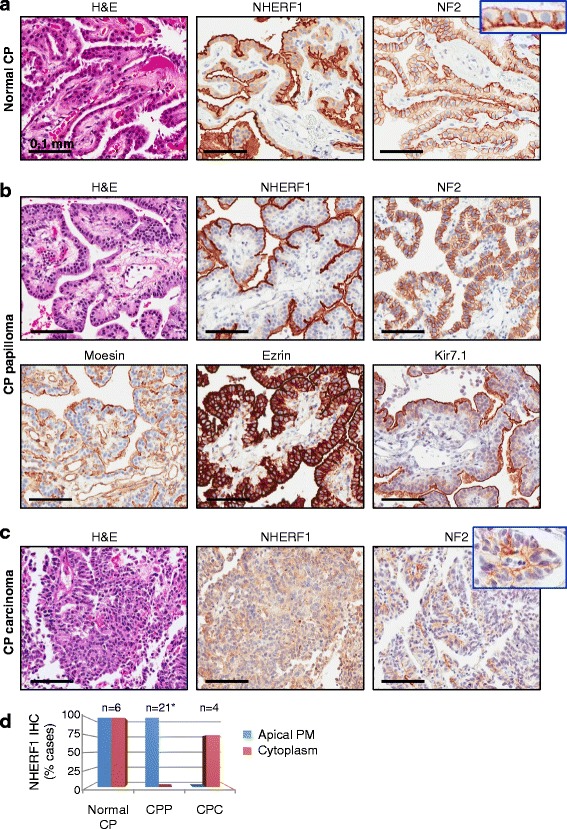
NHERF1 and ERM-NF2 expression in normal and transformed CP provides new diagnostic markers for CP tumors. **a** IHC with NHERF1 and NF2 antibodies in normal CP shows apical plasma membrane and cytoplasmic distribution of NHERF1 and characteristic basolateral plasma membrane expression of NF2. The inset shows additional faint apical plasma membrane expression of NF2. The H& E panels in A–C are shown for comparison. **b** IHC with indicated antibodies of CP papilloma shows prominent apical plasma membrane expression of NHERF1 that is more pronounced than the Kir7.1 labeling, basolateral plasma membrane expression of NF2, strong ezrin expression in the cytoplasm and at the apical plasma membrane and moesin labeling of the apical plasma membrane but also of the vasculature. **c** IHC with NHERF1 and NF2 antibodies in CP carcinoma (Case CPC3, Additional File 1: Table S1) shows delocalization to the cytoplasm of NHERF1 and focally preserved basolateral expression of NF2 (inset). **d** Quantification of the NHERF1 subcellular distribution in normal CP, CP papilloma (CPP) and carcinoma (CPC) cases. PM, plasma membrane. *From 22 cases of CPP, one had predominant oncocytic-like morphology and was not included in this analysis

To understand the role of NHERF1 and ERM-NF2 proteins in normal and transformed CP, we analyzed their expression by using specific antibodies. Because ot antibody cross-reactivity between the highly homologous proteins ezrin, radixin and moesin [4, 20], we used WB to test the specificity of several commercially available antibodies against control cell lysates following shRNA depletion of individual ERM proteins (Fig. 1). The selected specific antibodies revealed high expression levels of moesin, ezrin and NHERF1 in normal CP (Fig. 1). By IHC, a large fraction of NHERF1 was localized at the apical plasma membrane and the remainder in the cytoplasm in normal CP cells (Fig. 2a and Additional File 1: Figure S1). Moesin appeared only at the apical plasma membrane (Additional File 1: Figure S1) and ezrin displayed strong cytoplasmic and apical plasma membrane expression. NF2, another member of the ERM family with lower homology to the other members [4], was strongly expressed at the basolateral plasma membrane and less at the apical plasma membrane (Fig. 2a, inset, and Additional File 1: Figure S1). These findings show apical plasma membrane localization of NHERF1, ezrin and moesin in CP cells, similarly to other polarized epithelia where they play a structural role [8–10].

To assess the potential diagnostic use of NHERF1 and ERM-NF2 for CNS epithelial-like papillary tumors, we assembled a total of 43 papillary tumors, of which 32 cases were CP tumors from both adult and pediatric subjects (Table 1). These had a clear segregation based on location to the posterior fossa or lateral ventricles for the adult or pediatric populations, respectively, that was recently captured in molecular studies, as well [21]. When available, the clinical information for WHO grade II and III CP tumors and PTPR is also shown (Additional File 1: Table S1).

**Table 1:** NHERF1 and NF2 in the differential diagnosis of papillary tumors of the CNS

Diagnosis	Patients					IHC	
	No. cases Gender	Median age (range, yrs)	Location			NHERF1	NF2
			4^th^V	3^rd^V	LV		
CPP Adult	13 (6 F, 5 M)	43 (20–73)	12^1^	1	0	Apical PM	Basolateral and apical PM
CPP Pediatric	9 (4 M, 5 F)	0.5 (0.25-6)	1	2	6	Apical PM	Basolateral and apical PM
aCPP Adult	2 (1 M, 1 F)	40 (35, 46)	2	0	0	Cyt, variable^2^;	Basolateral and apical PM
aCPP Pediatric	4 (2 M; 2 F)	0.7 (0.3-1.6)	0	0	4	Cyt, apical PM	Basolateral and apical PM
CPC Adult	1 (1 F)	37	1	0	0	Cyt or absent	Basolateral and apical PM
CPC Pediatric	3 (2 M, 1 F)	1.7 (1, 2)	0	0	3	Cyt or absent	Basolateral and apical PM
PTPR	5 (3 M, 2 F)	40 (33–62)	0	5	0	Microlumens, apical PM	Cytoplasmic, membranous
Metastases^3^	6 (4 F, 2 M)	65 (43–73)				Apical PM	Absent, apical PM, nuclear

CPP, CP papilloma; aCPP, atypical CPP; CPC, CP carcinoma; M, male; F, female; 4^th^V, 4th ventricle; 3^rd^V, 3rd ventricle; LV, lateral ventricle; PM, plasma membrane; Cyt, cytoplasmic

^1^These 12 tumors were located in the posterior fossa, either in the 4th ventricle (6) or in the cerebello-pontine angle (6)

^2^In one atypical CPP case, NHERF1 apical PM staining as in CPP was seen in papillary regions, and rings and microlumens as in ependymoma were seen in confluent areas

^3^These metastases were selected based on papillary morphology. They include papillary thyroid carcinoma (3; frontal lobe, cerebellum, spinal cord), lung adenocarcinoma (2; parietal lobe) and serous ovarian carcinoma (1; temporal lobe)

Analysis of the NHERF1/ERM-NF2 IHC profile in CP papilloma (WHO grade I) showed similarities but also differences with normal CP (Fig. 2b and Additional File 1: Figure S1). NHERF1 was strongly expressed in CP papilloma, almost exclusively at the apical plasma membrane. The cytoplasmic levels were either very low or undetectable. A notable exception were the areas of oncocytic-like change in which NHERF1 and, as discussed below, NF2 staining was identical to that of normal CP (Additional File 1: Figure S2), suggesting perhaps an intermediate hyperplastic lesion. The oncocytic-like change was focal and present only in few cases of CP papilloma, except for one case in which it formed the bulk of a cystic tumor. The subcellular localization of moesin, ezrin and NF2 in CP papilloma was similar to that from normal CP (Fig. 2a–b and Additional File 1: Figure S1). The continuous apical plasma membrane staining of NHERF1 was reliably detected in all 22 cases of CP papilloma and showed more robust staining than that of the inward rectifier potassium channel Kir7.1 (Fig. 2b).

The analysis of 4 cases of CP carcinoma (WHO grade III) showed cytoplasmic expression of NHERF1 in three of four cases and loss of apical plasma membrane staining in all cases (Fig. 2c–d and Additional File 1: Figure S3). Importantly, compact areas with similar morphology from CP papilloma and carcinoma could be readily distinguished by NHERF1 IHC (Additional File 1: Figure S3). NF2 showed focal areas of basolateral plasma membrane staining in CP carcinoma (Fig. 2c and Additional File 1: Figure S3) that, as discussed below, is important in demonstrating the CP origin of the tumor.

**Fig. 3:**
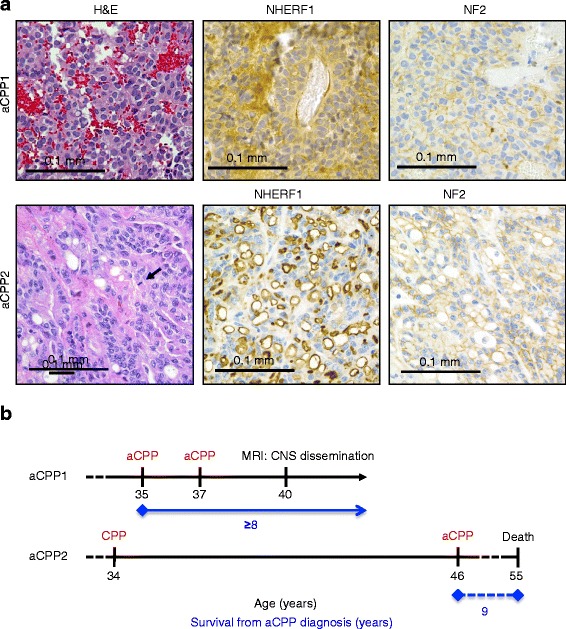
Clinico-pathologic correlates in two adult patients with atypical CP papilloma (aCPP). **a** Distinct patterns of NHERF1 IHC in two aCPP adult cases, labeling the cytoplasm, in aCPP1, and rings and microlumens in compact areas, in aCPP2. NF2 IHC has membrane staining in both cases, which helps cueing the CP origin of the tumors. The arrow indicates a mitotic figure in aCPP2. **b** Time course of clinical events shows disease onset, surgical interventions and pathologic diagnosis in red, and radiologic progression or death in black. The survival is shown in blue. aCPP1 is alive at the time of this analysis, with wide CNS dissemination, including the lumbar spinal cord. Note sequential progression from CP papilloma (CPP) to aCPP in aCPP2

Atypical CP papilloma (WHO grade II) is a rather controversial entity that is intermediary between the other two categories of CP tumors [21–23]. We studied two adult and four pediatric cases (Tabel 1 and Additional File 1: Table S1). The former were recurrent posterior fossa CP neoplasms with extensive areas of blurring of the papillary pattern, nuclear pleomorphism, necrosis or brain invasion, intermediate or high Ki67 proliferation index, but a mitotic count lower than 2 mitoses/10 high-power fields (Fig. 3a). In aCPP1, NHERF1 IHC showed cytoplasmic staining, similarly to CP carcinoma, and in aCPP2, apical plasma membrane staining in the papillary areas, and rings and microlumens, in the areas of compact growth (Fig. 3a). The pattern of NHERF1 staining in the compact growth areas of aCPP2 differed from the patterns seen in CP papilloma or carcinoma (Additional File 1: Figure S3), but resembled to that from ependymoma [8]. NF2 plasma membrane staining was present in both cases (Fig. 3a). Both adult patients had clinical progression of their disease (Fig. 3b and Additional File 1: Table S1). Two of the four pediatric atypical CP papilloma cases had scattered compact areas with loss of plasma membrane NHERF1 staining. Everywhere else, the morphology was papillary with an undistinguishable NHERF1 staining pattern from CP papilloma.

Resection specimens containing both normal CP and CP papilloma provided an opportunity to compare protein expression between non-neoplastic and neoplastic regions (Additional File 1: Figure S1). These were sometimes present on the same papillary trunk, showing continuity between the two forms (Additional File 1: Figure S4). We noted relatively decreased apical plasma membrane staining of NHERF1 and moesin in papilloma (Additional File 1: Figure S1). This suggests an attenuation of the density of microvilli at the apical plasma membrane in papilloma, without loss of cell polarity. The further loss of NHERF1 from the apical plasma membrane in carcinoma with either detectable or undetectable cytoplasmic expression (Fig. 2d) is compatible with loss of cell polarity, disorganized compact growth, and a more advanced stage of transformation. Although NF2 basolateral plasma membrane staining was preserved in normal CP and CP tumors, a decreased expression and a more focal distribution was observed with increasing tumor grade (Fig. 2c and Additional File 1: Figure S1). This tendency is most likely due to the known tumor suppressor function of NF2 [4, 13].

These results show that NHERF1 and NF2 staining patterns change along the CP normal to neoplastic progression spectrum. In particular, NHERF1 subcellular localization is very useful in differentiating CP papilloma with compact areas from tumors of higher grades.

### NHERF1 is a diagnostic marker for papillary tumors of the pineal region (PTPR)

**Fig. 4:**
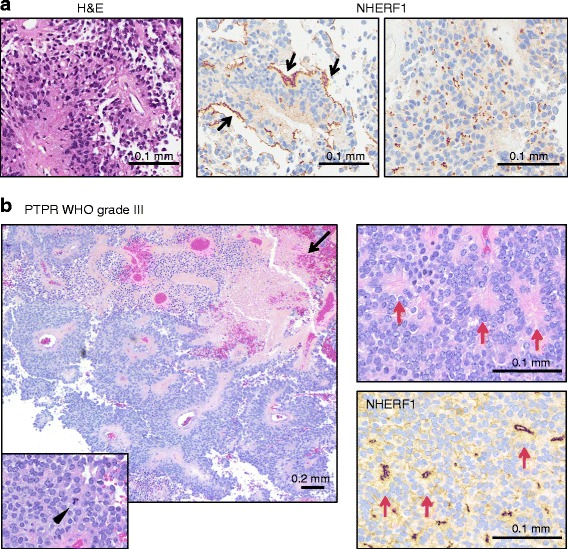
Mixed apical plasma membrane and microlumen NHERF1 labeling pattern in PTPR. **a** H& E of PTPR1 case shows typical multilayered papillary histology. IHC with NHERF1 antibody shows a characteristic mixed labeling pattern with focal apical plasma membrane staining (arrows) and microlumens (right panel). **b** H& E in PTPR4 shows densely cellular multi-layered papillae with areas of necrosis (arrow). Compact areas have many mitoses, including atypical ones (arrowhead in inset), and ependymal morphology, including true rosettes, also highlighted by NHERF1 IHC (red arrows)

We have recently reported that NHERF1 is associated with the specialized polarity structures from ependymoma and can be used as a reliable diagnostic marker in this tumor due to its high sensitivity for microlumen detection [8]. PTPR is defined as a neuroepithelial papillary mass arising posteriorily in the 3rd ventricle [24]. It has been postulated to originate from ependymal cells of the subcommissural organ [24, 25]. Due to an increased recurrence rate, PTPRs may correspond to WHO grades II or III. Among the five PTPR cases analyzed (Table 1 and Additional File 1: Table S1), one was diagnosed as WHO grade III based on a high number of mitoses. Architecturally, PTPR is formed by wide multi-layered papillae that frequently merge into compact growth (Fig. 4). Because PTPR shows microvilli at the apical plasma membrane by electron microscopy [25], we predicted that NHERF1 would highlight this specialized membrane. As predicted, NHERF1 IHC revealed apical plasma membrane staining (Fig. 4a, arrows). The staining pattern was focal, not continuous as in CP papilloma. Importantly, a microlumen NHERF1 pattern similar to the one in ependymoma [8] was evident in all PTPR cases (Table 1, Fig. 4a). Occasionally, punctate membrane staining was also present at the basal cell boundary at the interface with the vessels. NF2 was mostly cytoplasmic in compact areas and membranous in papillary areas (Additional File 1: Figure S5). Moesin had a strong continuous plasma membrane staining in the papillary areas in all the cases (Additional File 1: Figure S5), distinguishing PTPR from CP papilloma (Fig. 2b). The WHO III PTPR4 case had an overall Ki67 proliferation index of 10.6 % and a maximum of 75 %, necrosis and an increased number of mitoses, including atypical ones (Fig. 4b, left panel and inset). Although crowded, the nuclei were surprisingly monomorphic, round and with smooth contours. The papillary areas had a mixed NHERF1 pattern as in the other PTPR cases, showing microlumens and focal apical membrane staining. In compact areas, true rosettes were apparent and their lumens were labeled by NHERF1 as in ependymoma (Fig. 4b, left panels, and Additional File 1: Figure S6). Because of the advanced age of this patient, metastatic disease was ruled out by the panel of antibodies mentioned below for thyroid, lung and ovarian/uterine carcinomas and also by mammaglobin for breast cancer. Otherwise, this tumor was positive for Cam5.2 and MAP-2 and negative for EMA, GFAP and synaptophysin.

### NF2 expression distinguishes CP tumors from metastatic papillary carcinomas

**Fig. 5:**
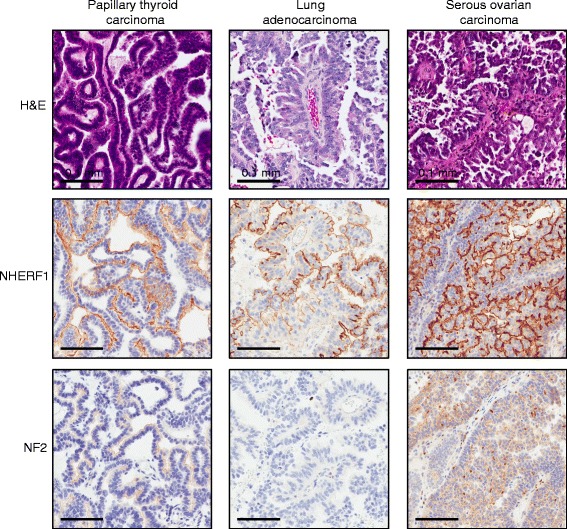
NHERF1 and NF2 expression in papillary metastatic carcinoma to the CNS. Representative cases of papillary thyroid carcinoma (67 year-old male), lung adenocarcinoma (71 year-old female), and serous ovarian carcinoma (43 year-old female) with papillary morphology assessed for IHC with NHERF1 and NF2 antibodies show apical plasma membrane localization of NHERF1 and a range of expression profiles for NF2. Note lack of NF2 basolateral plasma membrane staining in metastatic carcinomas, distinguishing these from CP tumors

Metastatic cancers to the CNS, especially carcinomas, may have a papillary architecture that elicits the differential diagnosis with CP tumors. We analyzed the expression of NHERF1 in 6 metastatic tumors with papillary architecture arising in various CNS locations, including posterior fossa (Table 1, Fig. 5). The three papillary thyroid carcinoma metastases were located in the right cerebellum, right frontal lobe and the epidural space of the spinal cord, the two lung adenocarcinoma metastases, in the right and left parietal lobes, and the serous ovarian carcinoma, in the right temporal lobe. The 6 papillary CNS metastases had a complete prior workup with IHCs confirming their origin, such as TTF1 and thyroglobulin for papillary thyroid carcinoma, TTF1, CK7, CK20 for lung adenocarcinoma, and Pax8, WT1, CK7, CK20 for serous carcinoma of the ovary. Apical plasma membrane NHERF1 labeling was present in all cases, either diffuse, as in the serous carcinoma of the ovary case and a subset of papillary thyroid carcinoma and lung adenocarcinoma, or focal, in the remainder of the cases (Fig. 5). When diffuse, this NHERF1 staining pattern was identical to that seen in CP papilloma, indicating that NHERF1 cannot differentiate between these tumors. NF2 staining in metastases consistently differed from CP tumors. While NF2 showed a reliable basolateral plasma membrane pattern in CP tumors, its expression in metastases was variable and ranged from apical to focal nuclear to absent (Fig. 5 and Table 1). Absent NF2 expression was noted in one lung adenocarcinoma case (shown in Fig. 5), and two papillary thyroid carcinoma cases. These results suggest that NF2 can be used to differentiate metastatic papillary carcinomas from CP tumors.

## Discussion

CP tumors are rare papillary neoplasms that present mostly in children, in approximately 80 % of cases. In contrast to CP papilloma for which total surgical resection is curative in the large majority of cases, CP carcinoma has an aggressive course requiring adjuvant therapy. The distinction between CP papilloma and carcinoma can be difficult, as areas of confluent growth and mitoses may be focal. In addition, routine IHC profiles do not differ significantly among the three grades of CP tumors. In particular, Kir7.1 has been shown to be a specific marker of CP papilloma [26]. However, its sensitivity is approximately 67 % [26], and its staining is not as robust as for NHERF1. We show here that NHERF1 IHC may aid distinguishing between CP papilloma and CP carcinoma. Loss of NHERF1 apical plasma membrane staining or enhanced cytoplasmic expression points to areas of compact growth and mitoses and marks a more aggressive phenotype with loss of membrane polarity. The cut off is not as clear in atypical CP papilloma, but an NHERF1 staining pattern similar to CP carcinoma or ependymoma favors a more aggressive phenotype and tumor recurrences. In the latter scenario, the differential diagnosis with ependymoma, especially the papillary variant, needs to be entertained, and a small biopsy containing only compact areas may be difficult to interpret.

NHERF1 IHC also shows a characteristic pattern in PTPR, with mixed microlumen and focal apical plasma membrane staining, and distinguishes this tumor from CP papilloma. It is also consistent with PTPR being intermediate in differentiation between ependymomas and CP tumors as previously suggested [27]. A recent study has shown that PTPR has a distinct molecular profile from ependymoma and CP papilloma [28]. Although many IHC markers have been studied, including EMA, cytokeratins, GFAP, MAP-2, and Kir7.1 [29, 30], none show a diagnostically useful, reliable staining profile. The location of the tumor, papillary architecture, and presence of microlumens by NHERF1 IHC offer the best clues to the diagnosis of this entity. Testing for NHERF1 IHC of a large series of other CNS neoplasms that could arise in any location, including the pineal gland, did not detect microlumens in 22 cases of WHO grade II and III diffuse gliomas, 4 cases of pilocytic astrocytomas and 2 cases of atypical teratoid/rhabdoid tumors [8].

While metastatic disease to the CNS usually affects an older population than CP tumors and the history of a primary tumor may be known, diagnostic uncertainties between papillary metastasis and CP tumors do occur. We have found that NF2 is a marker for CP with a characteristic subcellular localization at the basolateral plasma membrane in normal or transformed CP. This subcellular localization was not detected in metastatic papillary carcinomas and may thus be used to differentiate between CP tumors and metastatic carcinomas. Moreover, whereas all 32 choroid plexus tumors in this series expressed NF2, three out of six papillary metastatic tumors were negative for NF2. Loss of chromosome 22 locus for *NF2* is a frequent event in papillary thyroid carcinoma [31], explaining that two such tumors lacked NF2. *NF2* mutations, albeit rare, were reported in lung adenocarcinoma, as well [32].

Similar progressive NHERF1 changes as in CP tumors, with loss of apical plasma membrane expression and cytoplasmic accumulation have been noted previously in the colorectal cancer transformation sequence [14]. It is tempting to propose that a normal - > papilloma - > carcinoma transformation sequence takes place for CP tumors as well. Due to the rarity of CP tumors, only few documented cases of CP tumor histologic progression are reported in the literature [33, 34]. An additional case of worsening histological disease is the aCPP2 case in our study. Intriguingly, a high proportion of these progressive cases fall into the adult rather than the pediatric population. Recent molecular profiling studies segregated CP carcinomas from CP papillomas, and adult from pediatric disease [21–23]. However, these studies did not address the possibility of progressive disease. Additional investigations are required to delineate the full spectrum of CP tumors, and delineate prognostic markers aiming patient stratification for clinical management.

## Conclusion

NHERF1 and NF2 IHC is diagnostically useful for papillary tumors of the CNS. NHERF1 emerges as a marker of CP tumors based on its structural role in specialized epithelia. NHERF1 has been shown to exert a tumor suppressor function when localized at the plasma membrane [14, 18], and perhaps loss of plasma membrane localization permits oncogenic progression in CP tumors. Moreover, an oncogenic function of NHERF1 in the cytoplasm has been proposed in both colorectal and melanoma cells [14, 35]. As therapeutic efforts are underway to inhibit the association of NHERF1 to its PDZ-domain ligands [36, 37], its role needs to be further investigated in this lethal childhood malignancy.

## Abbreviations

C, carboxyl; CNS, central nervous system; CP, choroid plexus; CSF, cerebrospinal fluid; ERM, ezrin-radixin-moesin; IHC, immunohistochemistry; N, amino; NF2, neurofibromin 2; NHERF1/EBP50, Na/H exchanger regulatory factor 1/ ERM-binding phosphoprotein 50; PDZ, PSD95-Dlg1-ZO1; PTPR, papillary tumor of the pineal region; WB, Western blot; WHO, World Health Organization.

## Additional file

Additional file 1: Table S1. Clinical correlates for WHO II and III CP tumors and PTPR. **Figure S1.** Comparison between the expression of NHERF1, moesin and NF2 in normal CP and CP papilloma (CPP). IHC with indicated antibodies of serial sections from a resection specimen (1 year-old female) containing both normal CP (right) and CP papilloma (left). Note higher expression of all 3 proteins in normal CP than in papilloma and cytoplasmic NHERF1 expression in normal CP but not in CP papilloma. **Figure S2.** The staining profiles of CP papilloma with oncocytic-like change and normal CP are similar. IHC with indicated antibodies of serial sections (×20 original magnification) from a resection specimen (22 year-old male) containing CP papilloma with (left) or without (upper right) oncocytic-like change. **Figure S3.** NHERF1 IHC distinguishes compact areas in CP papilloma (CPP) and carcinoma (CPC). Compact areas may show ill-defined papillary architecture in cases of CPP (6 year-old female) and CPC (1 year-old male). NHERF1 preserves the characteristic IHC pattern in CPP and shows faint cytoplasmic and focal membranous staining in this CPC example with extensive necrosis. Also shown are NF2 membrane staining and mitoses (arrows) (insets, 40× original magnification). **Figure S4.** NHERF1 IHC highlights normal CP in a resection speciment of CP papilloma. Serial sections (×10 original magnification) from a resection specimen (5 month-old female) containing both normal CP (right) and CP papilloma (left) show clear demarcation by NHERF1 IHC. Note also continuity of normal CP, at the tip, and CP papilloma, at the base, of the same major papillary stem. **Figure S5.** NF2 IHC in compact and papillary areas from CP papilloma (CPP) and PTPR, as compared to ependymoma. IHC with NF2 antibody shows distinct basolateral and apical plasma membrane staining in compact and papillary areas of CPP (same case as in Fig. S3). NF2 is cytoplasmic in compact areas from PTPR and membranous in papillary areas. Strong plasma membrane moesin staining is also noted in papillary areas in PTPR. NF2 is cytoplasmic in ependymoma. Very faint NF2 membranous staining of polarized structures may be seen in PTPR (rosette lumens in this example) or ependymoma (microlumens and rings). In the example of ependymoma, the same area in a serial section stained with NHERF1 antibody shows rings and microlumens. **Figure S6.** Example of area with rosettes in ependymoma (WHO grade II). H&E and NHERF1 IHC of serial sections from a resection specimen (5 month-old female) showing labeling of rosette lumens (arrows) and of microlumens. (PDF 1266 kb)
