# Supplementary material for: NHERF1/EBP50 and NF2 as diagnostic markers for choroid plexus tumors
Source: Acta Neuropathol Commun. 2016 May 27;4:55. doi: 10.1186/s40478-016-0329-0 (PMC4882843; doi:10.1186/s40478-016-0329-0)
Supplement: Additional file 1: Table S1. — Clinical correlates for WHO II and III CP tumors and PTPR. Figure S1. Comparison between the expression of NHERF1, moesin and NF2 in normal CP and CP papilloma (CPP). IHC with indicated antibodies of serial sections from a resection specimen (1 year-old female) containing both normal CP (right) and CP papilloma (left). Note higher expression of all 3 proteins in normal CP than in papilloma and cytoplasmic NHERF1 expression in normal CP but not in CP papilloma. Figure S2. The staining profiles of CP papilloma with oncocytic-like change and normal CP are similar. IHC with indicated antibodies of serial sections (×20 original magnification) from a resection specimen (22 year-old male) containing CP papilloma with (left) or without (upper right) oncocytic-like change. Figure S3. NHERF1 IHC distinguishes compact areas in CP papilloma (CPP) and carcinoma (CPC). Compact areas may show ill-defined papillary architecture in cases of CPP (6 year-old female) and CPC (1 year-old male). NHERF1 preserves the characteristic IHC pattern in CPP and shows faint cytoplasmic and focal membranous staining in this CPC example with extensive necrosis. Also shown are NF2 membrane staining and mitoses (arrows) (insets, 40× original magnification). Figure S4. NHERF1 IHC highlights normal CP in a resection speciment of CP papilloma. Serial sections (×10 original magnification) from a resection specimen (5 month-old female) containing both normal CP (right) and CP papilloma (left) show clear demarcation by NHERF1 IHC. Note also continuity of normal CP, at the tip, and CP papilloma, at the base, of the same major papillary stem. Figure S5. NF2 IHC in compact and papillary areas from CP papilloma (CPP) and PTPR, as compared to ependymoma. IHC with NF2 antibody shows distinct basolateral and apical plasma membrane staining in compact and papillary areas of CPP (same case as in Fig. S3). NF2 is cytoplasmic in compact areas from PTPR and membranous in papillary areas. Strong plasma membrane moesin [file 40478_2016_329_MOESM1_ESM.pdf]

Supplemental Table 1. Clinical correlates for WHO II and III CP tumors and PTPR.

Diagnosis (WHO grade)	Sex; age	Location	Surgery (total/partial)	Treatment	Progression	
					Radiologic	Clinical
aCPP1 (II)	F35	4th V	01/2008	2 yr PO RT	2 yr PO[1]	Alive > 8 yr PO
aCPP2 (II)	M46	4th V	04/2004	9 yr PO Chemo&RT	Yes	Death 9.3 yr PO
aCPP3 (II)	F1.7	R LV	No data	No data	No data	No data
aCPP4 (II)	M0.4	L LV	No data	No data	No data	No data
aCPP5 (II)	F0.3	R LV	No data	No data	No data	No data
aCPP6 (II)	M0.9	LV	No data	No data	No data	No data
CPC1 (III)	F37	PF	11/1999 (TR)	PO Stereotactic RT	1.1 yr PO[1]	Death 5 yr PO
CPC2 (III)	M1	R LV	2006	PO Chemo & RT	Yes	Regression[2]
CPC3 (III)	M2	R LV	2006 (TR)	1 yr PO Chemo[3]	3 yr PO[1]	Hospice 6 yr PO
CPC4 (III)	F1.7	R LV TL	08/2014 (TR)	PO Chemo & RT	0.3 yr PO[1]	Death 0.5 yr PO
PTPR1 (II-III)	M33	3rd V	04/2008 (TR)	3 yrs PO (γ-knife)	1 yr PO	>5 yr PFS since
PTPR2 (II-III)	F59	3rd V	10/2013 (PR)	PO IMRT	No	Stable
PTPR3 (II-III)	M37	3rd V	03/2014 (PR)	No	0.7 yr PO	Regression[2]
PTPR4 (III)	F62	3rd V	10/2014 (TR)	No	1.5 yr PO	Too short FU
PTPR5 (II-III)	M40	3rd V	06/2015 (PR)	No	No	Regression[2]

aCPP, atypical CP papilloma; CPC, CP carcinoma; M, male; F, female; 4thV, 4th ventricle; PF, posterior fossa; 3rdV, 3rd ventricle; LV, lateral ventricle; TL, temporal lobe; TR, total resection; PR,

partial resection; PO, post-operatory; Chemo, chemotherapy; IMRT, intensity-modulated radiation therapy; PFS, progression-free survival; FU, follow-up

[1]All these patients had dropdown metastases in the spinal cord at different levels. aCPP1, CPC1 and CPC3 had also recurrences in the tumor bed or in other ventricular locations, as well, with surgical resection for aCPP1 and CPC3.

[2]These enhancing nodules shrunk after being larger at previous MRI.

[3]This patient had multiple recurrences at 3 and 5 yr PO, for which he received resection and radiotherapy (3 yr PO) and radiotherapy and chemotherapy (5 yr PO).

SUPPLEMENTAL FIGURES

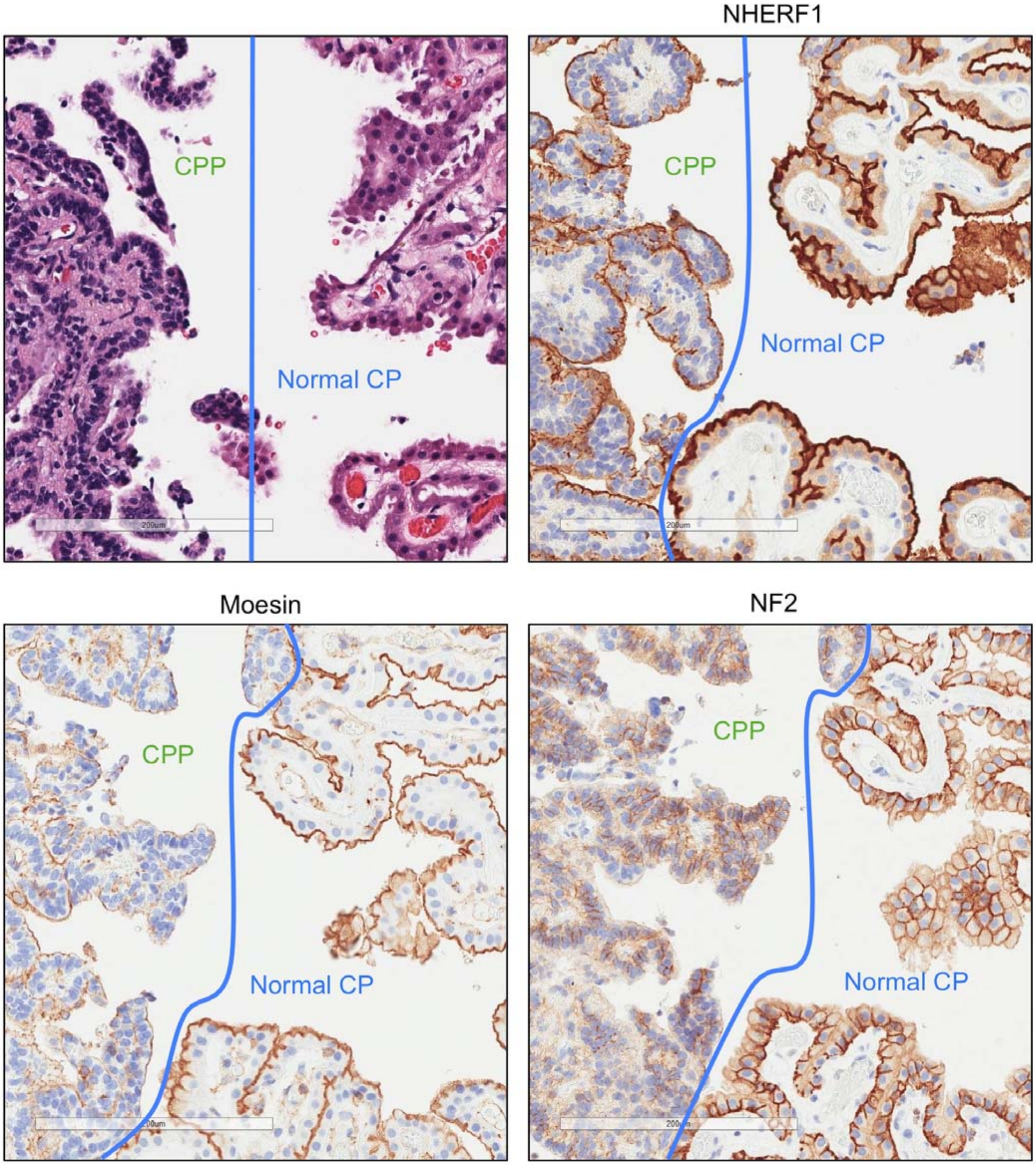

Figure S1. Comparison between the expression of NHERF1, moesin and NF2 in normal CP and CP papilloma (CPP). IHC with indicated antibodies of serial sections from a resection specimen (1 year-old female) containing both normal CP (right) and CP papilloma (left). Note higher expression of all 3 proteins in normal CP than in papilloma and cytoplasmic NHERF1 expression in normal CP but not in CP papilloma.

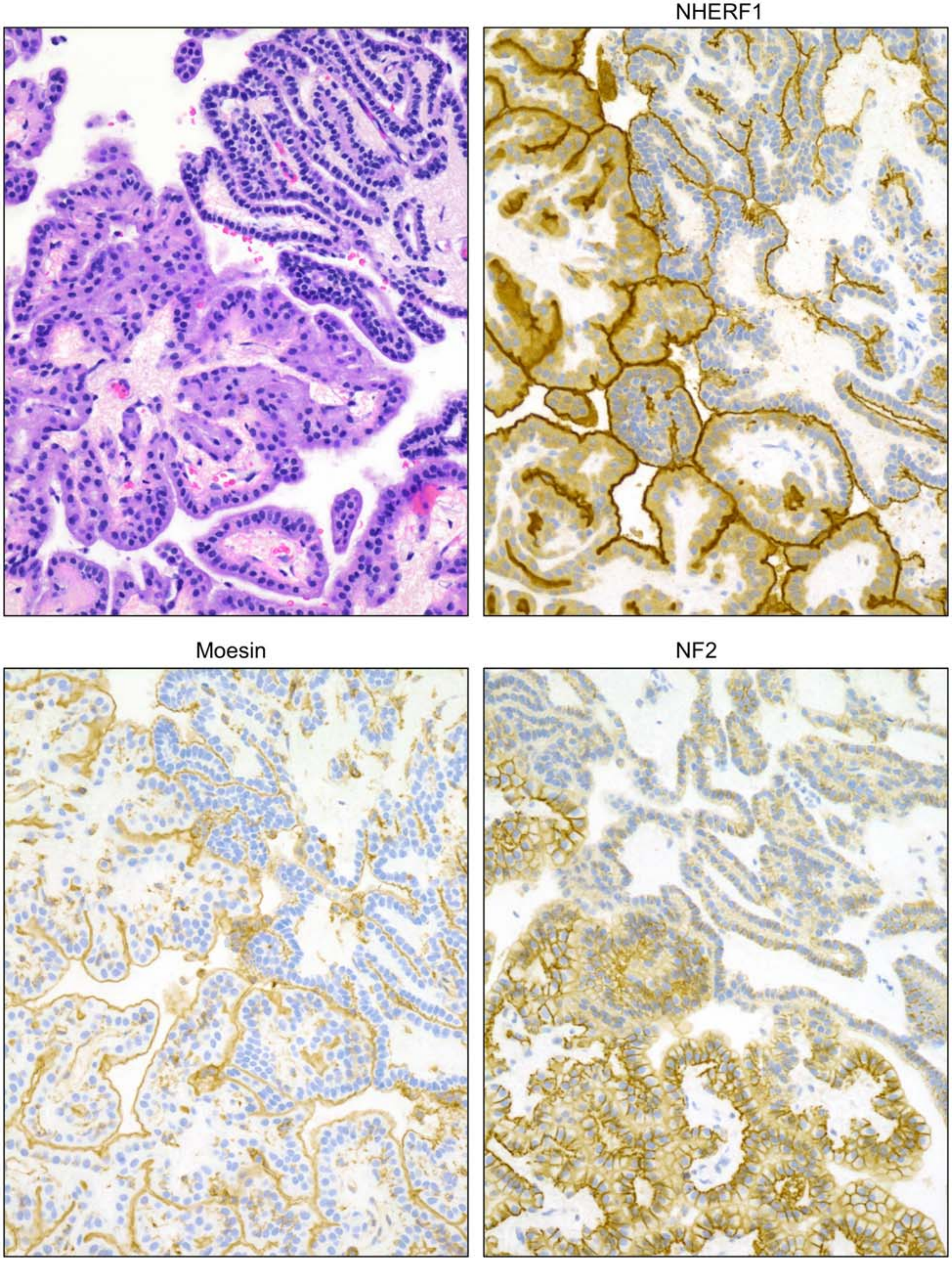

Figure S2. The staining profiles of CP papilloma with oncocytic-like change and normal CP are similar. IHC with indicated antibodies of serial sections (x20 original magnification) from a resection specimen (22 year-old male) containing CP papilloma with (left) or without (upper right) oncocytic-like change.

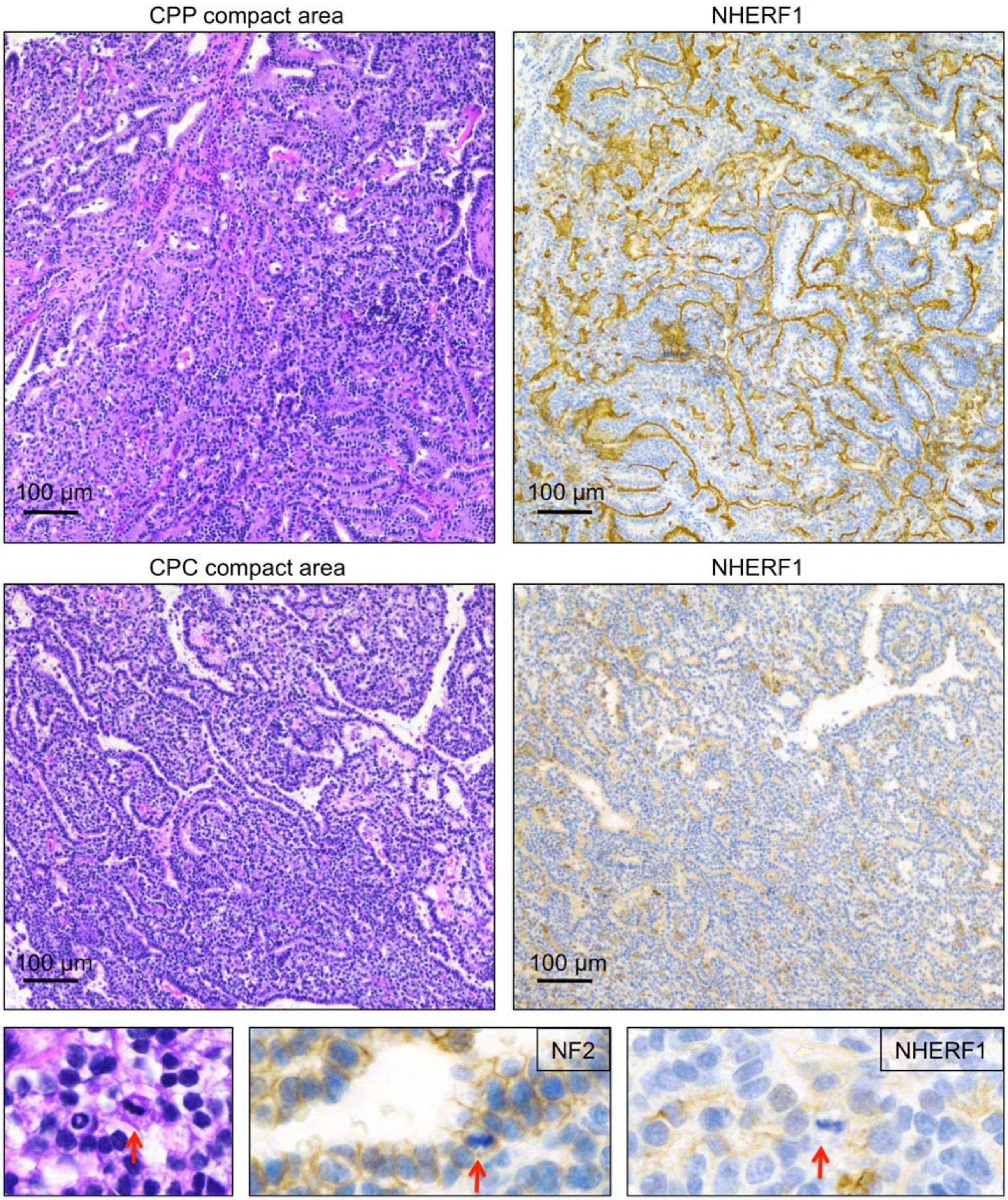

Figure S3. NHERF1 IHC distinguishes compact areas in CP papilloma (CPP) and carcinoma (CPC). Compact areas may show ill-defined papillary architecture in cases of CPP (6 year-old female) and CPC (1 year-old male). NHERF1 preserves the characteristic IHC pattern in CPP and shows faint cytoplasmic and focal membranous staining in this CPC example with extensive necrosis. Also shown are NF2 membrane staining and mitoses (arrows) (insets, 40x original magnification).

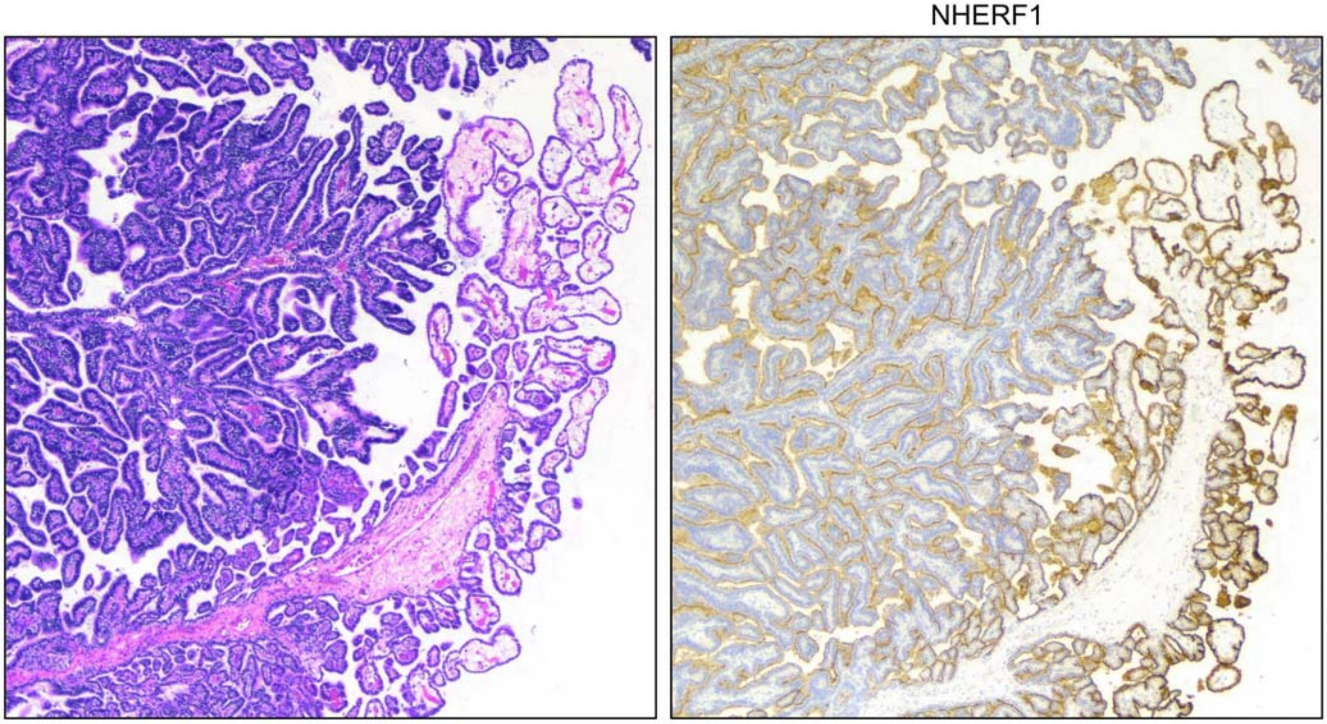

Figure S4. NHERF1 IHC highlights normal CP in a resection speciment of CP papilloma. Serial sections (x10 original magnification) from a resection specimen (5 month-old female) containing both normal CP (right) and CP papilloma (left) show clear demarcation by NHERF1 IHC. Note also continuity of normal CP, at the tip, and CP papilloma, at the base, of the same major papillary stem.

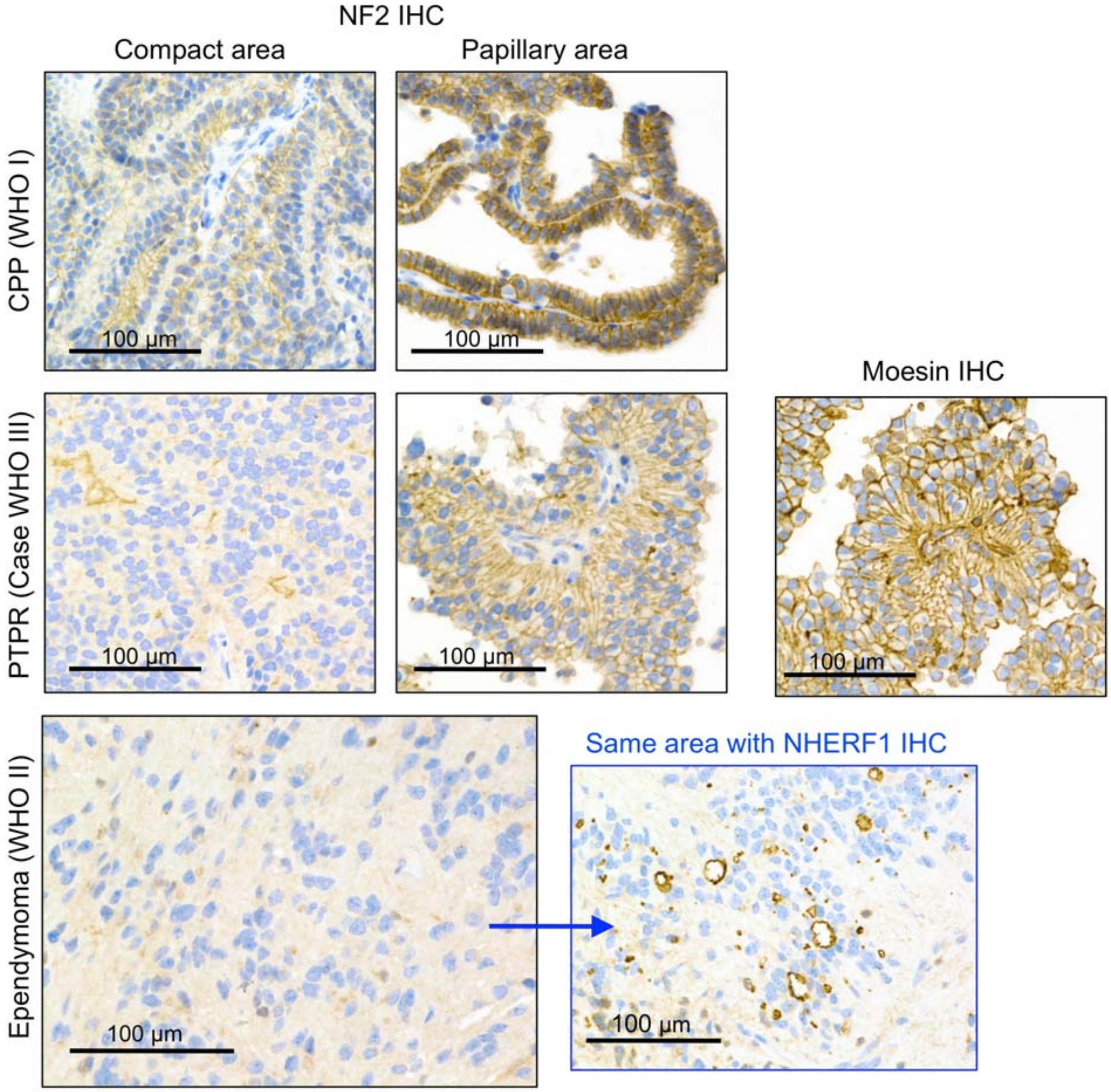

Figure S5. NF2 IHC in compact and papillary areas from CP papilloma (CPP) and PTPR, as compared to ependymoma. IHC with NF2 antibody shows distinct basolateral and apical plasma membrane staining in compact and papillary areas of CPP (same case as in Fig. S3). NF2 is cytoplasmic in compact areas from PTPR and membranous in papillary areas. Strong plasma membrane moesin staining is also noted in papillary areas in PTPR. NF2 is cytoplasmic in ependymoma. Very faint NF2 membranous staining of polarized structures may be seen in PTPR (rosette lumens in this example) or ependymoma (microlumens and rings). In the example of ependymoma, the same area in a serial section stained with NHERF1 antibody shows rings and microlumens.

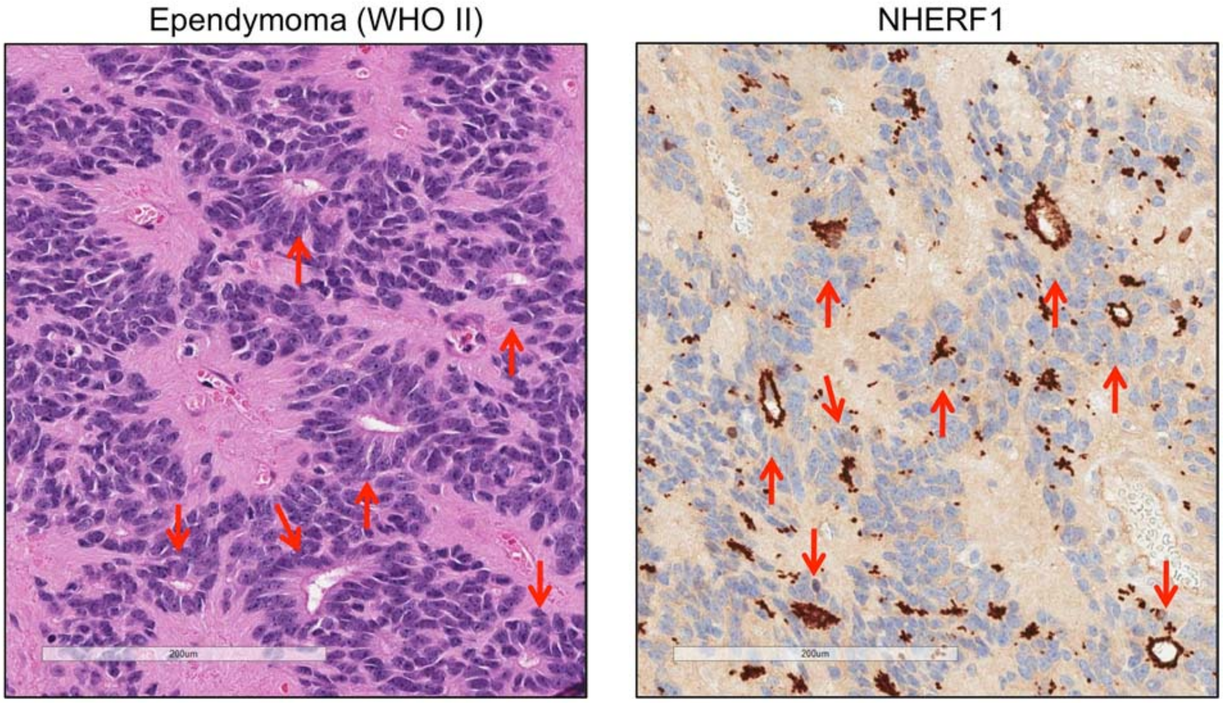

Figure S6. Example of area with rosettes in ependymoma (WHO grade II). H&E and NHERF1 IHC of serial sections from a resection specimen (5 month-old female) showing labeling of rosette lumens (arrows) and of microlumens.
